# Evaluation of the reboot coaching workshops among urology trainees: A mixed method approach

**DOI:** 10.1002/bco2.249

**Published:** 2023-05-02

**Authors:** Tmam Al‐Ghunaim, Judith Johnson, Chandra Shekhar Biyani, Rebecca Coleman, Ruth Simms‐Ellis, Daryl B. O'Connor

**Affiliations:** ^1^ School of Psychology University of Leeds Leeds UK; ^2^ Bradford Institute for Health Research Bradford Royal Infirmary Bradford UK; ^3^ School of Public Health and Community Medicine University of New South Wales Sydney Australia; ^4^ Department of Urology St James's University Hospital Leeds UK

**Keywords:** burnout, confidence, depression, Reboot‐C, resilience, urology trainees

## Abstract

**Background:**

Urology trainees experience high burnout, and there is an urgent need for acceptable and effective interventions. The current study evaluated Reboot coaching workshops (Reboot‐C), a tailored intervention based on cognitive–behavioural principles, with urology trainees.

**Objective:**

Our primary objective was to evaluate the acceptability of Reboot‐C among urology trainees. In addition, this study aimed to investigate whether there were changes in confidence, resilience, depression and burnout levels.

**Materials and method:**

A single‐arm design was used, including pre‐ and post‐online questionnaires and semi‐structured interviews.

**Result:**

Twenty‐one urology trainees replied to the survey, attended both Reboot‐C workshops and responded to the post‐intervention questionnaire. Thirteen of 21 (61%) urology trainees participated in the interview. Participating in Reboot‐C was associated with significant improvements in resilience and confidence and a significant reduction in burnout. However, there was no significant reduction in depression. Qualitative data indicated that Reboot was acceptable and helped participants develop useful skills.

**Conclusion:**

These findings pave the way for more conclusive studies on the efficacy of Reboot‐C for surgeons.

## 1 INTRODUCTION

The COVID‐19 pandemic caused detrimental physical and emotional impacts on healthcare professionals (HCPs),^1^ resulting in increased stress, worry, depressive symptoms and insomnia.^2^ According to the General Medical Council survey,^3^ 44% of UK trainee doctors suffered from burnout during the COVID‐19 pandemic, the highest rate on record. There is evidence to suggest that surgeons may be at higher risk for burnout than doctors from other specialties.^4^, ^5^ Some groups of surgeons suffer more than others; for example, urology trainees have been found to have one of the highest rates of burnout.^6^ According to a 2013 Medscape poll, 41% of urologists said they felt burnout; by 2021, that number had risen to 49%.^7^, ^8^ As such, there is an urgent need for supportive interventions for surgical trainees.

The reasons for burnout in urology trainees can be ascribed to a variety of issues relating to the nature of their work. According to Al‐Ghunaim et al. (2022), there are various reasons why surgeons suffer from burnout, including interpersonal conflict at work, demands greater than resources, the issue of work‐life balance and the devastating consequences of errors and poor patient outcomes.^9^ Stress, burnout and depression have both organisational and personal impacts. For example, stress and burnout have been found to contribute to high levels of surgeons turnover.^9^, ^10^ According to a recent report by the General Medical Council (GMC, 2021), 23% of physicians planned to leave the profession.^3^ Shanafelt et al. study of 7905 surgeons discovered that an error in the previous 3 months had a large, statistically significant negative effect on burnout (emotional tiredness, depersonalization and personal accomplishment).^11^ Whereas a recent qualitative study found that some surgeons quit their careers or left the country they were working in because of burnout.^9^ Therefore, burnout and mental health issues should be prioritised for research and intervention. Hence, it can be said that poor patient results can lead to surgeon burnout, and surgeon burnout might, in turn, cause them to leave their profession.

In terms of personal impacts, stress and burnout can lead surgeons to engage in harmful practices, including substance abuse.^9^ Burnout has also been shown to be related to a greater probability of reporting a mental health issue^12^ and higher levels of depression and anxiety.^13^ Moreover, another study found that the emotional exhaustion component of burnout was associated with increased suicide risk among surgeons.^14^

There are three main ways to reduce work stress: (1) lowering work demands, (2) increasing autonomy and (3) making the workplace more supportive.^15^ Some commentators have suggested that organisation‐directed interventions that aim to reduce demands and increase autonomy are the best way to reduce stress.^16^ While it is ideal and ethical to target primary sources of stress at the organisational level, such as reducing demands and increasing autonomy, this may be difficult to achieve in healthcare.^17^ Furthermore, evidence suggests that workplace support interventions like training, counselling and assistance programmes can have a significant impact on reducing burnout in physicians^18^ and may be valuable in improving staff wellbeing. The most crucial consideration may relate to the form supportive interventions are delivered in, as many existing psychological interventions have been criticised for taking a generic, ‘off‐the‐shelf’ approach not tailored to clinicians.^19^ To overcome this, there is a need for interventions that address the specific stressors which contribute to surgeons' burnout, such as poor patient outcomes, difficult interactions with colleagues and adverse events,^20^ yet no prophylactic intervention to prepare surgeons for these events has been evaluated.

One potentially relevant intervention that helps prepare clinicians for stressful healthcare events such as medical errors is Reboot (Recovery Boosting Training).^19^ Reboot is a training programme based on Cognitive Behaviour Therapy principles, which helps prepare health professionals for the emotionally taxing clinical situations they face during their work and which organisational measures cannot address,^21^, ^22^ such as untimely patient deaths, breaking bad news to patients, dealing with agitated or combative patients and being involved in adverse events.^23^ Reboot has been associated with reductions in burnout and depression and increases in confidence and resilience in a range of multidisciplinary HCPs and students.^23^

Another common criticism of psychological support interventions is that they are overly time‐resource intensive, making them too expensive for training courses to offer or for individuals to access.^24^ The standard Reboot format involves both two small‐group workshops and a 1‐hour, one‐to‐one coaching call,^23^ which may be viewed by some courses as too intensive or time‐demanding to offer to surgical trainees. No study has yet examined whether Reboot is still effective when only the workshops are offered, without the coaching phone calls. The present study focuses on evaluating Reboot coaching workshops (Reboot‐C), an adapted version of Reboot that involves the workshops alone, without a one‐to‐one coaching call.

To fill these gaps, the current study evaluated Reboot‐C in urology trainees. Our primary research question focused on determining whether Reboot‐C was acceptable to urology trainees. Secondary research questions explored whether there were changes in scores of key outcome measures (confidence, resilience, depression and burnout).

## 2 MATERIALS AND METHODS

### 2.1 Study design and setting

The study used a mixed method approach to data collection. A single‐arm pre‐post design using online questionnaires and telephone/online interviews was used. Recruitment and programme delivery were conducted over 4 months (June to September 2022). The study received ethical approval from the University of Leeds, School of Psychology Ethics Committee (PSYC‐508, date: 10/02/2021).

### 2.2 Participants and inclusion/exclusion criteria

Eligible participants were urology trainees in the UK. To guarantee that participants remained anonymous and information was not shared with employers, participants were not requested to provide identification or proof of employment. Participants responded to Twitter ads/study flyers to enrol in Urology Bootcamp 2022. The bootcamp is a 5‐day programme to train trainee surgeons in key skills.^25^ Those enrolled on Bootcamp 2022 were subsequently informed about Reboot‐C dates and the number of available cycles through emails. On the sign‐up form, participants could choose from three available Reboot‐C cycles.

### 2.3 Background to Reboot‐C

Reboot‐C uses evidence‐based cognitive–behavioural techniques to develop those characteristics known to confer psychological resilience in the face of errors or mistakes.^19^ Reboot‐C has the following three objectives: (1) to develop more flexible thinking, including normalising stress and failure and understanding the interactions between behaviour, mood and cognition; (2) to increase self‐esteem when experiencing stress and failure; and (3) to develop a better explanatory style to assist individuals in identifying and implementing more helpful personal habits in the context of stress and failure.^19^

Reboot was originally designed so that the materials could be tailored to each disciplinary group to which it was delivered. A Clinical Psychologist (JJ) and a Urology Consultant (SB) customised the materials for urology trainees (CSB). A Clinical Psychologist (JJ) and CBT Therapist (RC) led the workshops.

### 2.4 Evaluation overview

The pre‐ and post‐surveys included questionnaires (see supporting information Appendix S1) that assessed (1) confidence in coping with adverse events (‘confidence’), (2) resilience, (3) burnout and (4) depression. At the follow‐up time point, there was also a feedback questionnaire, and participants were invited to a subsequent interview with the researcher. This interview was conducted over a video platform or phone call, had a duration of around 30 min and was completed 1–2 weeks after the intervention.

### 2.5 Measures

We recorded the number of urology trainees who participated and were retained in the study (those who completed baseline questionnaires and then completed both workshops and follow‐up questionnaires/interviews) to indicate acceptability.

#### 2.5.1 Demographic questions

Participants provided demographic details, including their age, gender, ethnicity, stage of training and how many years of experience they had as surgeons.

#### 2.5.2 Confidence

This questionnaire had three items and a 4‐point response scale from ‘No, not at all’ to ‘Yes, definitely’. Possible scores ranged from 3 to 12. Higher scores reflect higher levels of confidence. The scale has been used in previous evaluations of Reboot.^19^, ^23^ In the current investigation, this scale's internal consistency was good (pre‐workshops, *α* = 0.85; post‐workshops, *α* = 0.88).

#### 2.5.3 Resilience

The Brief Resilience Scale (BRS), which evaluates how robust a person views themselves regarding adversity, consists of six items.^26^ Higher scores indicate greater resilience. The scale has a maximum possible score of 30 (range 6–30), and responses are given on a 5‐point Likert scale from ‘Strongly disagree’ to ‘Strongly agree’. In this study, the internal consistency of this scale was good (pre‐workshops: *α* = 0.78; post‐workshops: *α* = 0.86).

#### 2.5.4 Burnout items

The Oldenburg Burnout Inventory (OLBI) was utilised in a condensed form. The OLBI items with the three highest factor loadings on their respective subscales were kept in this version.^27^ The six statements had a response scale from 1 to 4, ‘Strongly Agree’ to ‘Strongly Disagree’. Possible scores ranged from 4 to 24. Higher scores reflect higher levels of burnout. The internal consistency of this scale was good in the present study (pre‐workshops: *α* = 0.77; *α* = post‐workshops: *α* = 0.80).

#### 2.5.5 Patient Health Questionnaire (PHQ‐9)

The PHQ‐9 is a validated tool for detecting depression and its severity. Its nine items are in line with the DSM‐IV criteria for diagnosing depression, and the scoring range is from 0 (not at all) to 3 (nearly every day). Total scores range from 0 to 27, with higher scores indicating more severe depression This scale's internal consistency was good in this study (pre‐workshops: *α* = 0.92; post‐workshops: *α* = 0.98).

#### 2.5.6 Feedback questionnaire

The feedback questionnaire was collected immediately after the second workshop. The questionnaire includes eight items that have been used in previous studies.^23^, ^28^ For the first four items, responses were given on a 5‐point Likert scale from ‘strongly disagree’ to ‘strongly agree’; higher scores indicate a more positive appraisal of the intervention (Table 2). Four more items allowed free‐text responses to ‘yes’ or ‘no’ questions.^19^

### 2.6 Interview

The first part of the interview focused on the trainees' perspectives on resilience and error management and how the workshops may have helped their resilience. The second part of the interview focused more specifically on their experiences with Reboot‐C (supporting information Appendix S2). To generate unanticipated insights, the thematic analysis was used to analyse all of the interview data.^29^ Thematic analysis was used to analyse all of the interview data because of its theoretical flexibility and ability to generate unexpected insights by providing a detailed account of the data.^30^

### 2.7 Procedures and data collection

This preliminary study did not aim to generate conclusive results regarding the effectiveness of the intervention. Instead, it can be regarded as an initial proof‐of‐principle study. As such, our intended sample size was smaller, based on what was needed to generate useful qualitative data to inform the acceptability and potential usefulness of the intervention in this group.^31^ Thus, we aimed to recruit 20 participants.

Study information was sent to the trainees who registered for Urology Bootcamp 2022. Deanery tutors and administrators sent this information by email. To participate, they were asked to contact the research team. Online posters were promoted on Twitter using the hashtag #urology #UK #NHS to recruit urology trainees who did not attend the Urology Bootcamp 2022. Urology trainees interested in participating in Reboot‐C were emailed by the research team with more information. Those who wanted to participate were provided with a link to the secure online server Qualtrics, where they completed informed consent and baseline questionnaires. After that, they were provided with details about the online workshops. The participants answered the questionnaires and attended the first online workshop (2 h). After attending both workshops, they were then invited to complete the questionnaires again post‐intervention. They were also invited to complete a phone/video interview after the workshops. Sound and video files were uploaded to secure University of Leeds One Drive servers (password protected) within 24 h. Any identifiable information was anonymised during transcription, for example, by removing all names of places and people.

### 2.8 Statistical analysis

To determine whether there was a significant difference between the confidence, resilience, burnout and PHQ‐9 questionnaire scores before and after attending the workshops, paired *t*‐tests with bootstrapping were utilised. Using SPSS to analyse the data, the significance threshold was determined at 0.05.

#### 2.8.1 Data analysis for the interview

This study utilised Braun and Clark's five‐step paradigm for thematic analysis.^29^ TG, a psychology PhD candidate, and JJ, a psychology academic and Clinical Psychologist, independently coded two transcripts and compared their results to facilitate triangulation of analysis. The remaining transcripts were coded by TG. The themes were then double‐checked and refined during meetings with the entire research team (TG, JJ, DOC, SB).

## 3 RESULTS

### 3.1 Descriptive analysis

We recruited a total of 26 urology trainees who attended the first workshop; of those, 23 (88.5%) participated in the second workshop. The low dropout indicates acceptability.^32^ Two participants were removed from the final analysis: the first, who did not respond to the initial survey but attended both workshops and completed the follow‐up survey, and the second, who answered the first survey and participated in both workshops but did not answer the follow‐up survey. Twenty‐one urology trainees responded to the survey, attended both Reboot‐C workshops and answered follow‐up questions (see Table 1), and 13 urology trainees attended the interview.

**TABLE 1:** Demographic questions.

Age	11 (52.4%) participants were aged 21 to 31 10 (47.6%) were aged 31 to 40
Gender	13 (61.9%) were men. 8 (38.1%) were women.
The ethnic background	4 (19%) were White British. 1 (4.8%) was Caribbean. 4 (19%) were African. 6 (28.5%) were Indian. 1 (4.8%) was Arab. 2 (9.5%) were White and Black Caribbean. 2 (9.5%) were from other White backgrounds. 1 (4.8%) preferred not to say.
Training stage	ST3 5 (23.8%) ST3 8 (38.1%) ST4 4 (19%) ST5 2 (9.5%) ST7 1 (4.8%) 1 (4.8%) other as a Clinical Development Fellow.
Practising surgeons	2 (9.5%) had 2 years of experience. 3 (14.3%) had 3 years of experience. 3 (14.3%) had 4 years of experience. 5 (23.8%) had 5 years of experience. 4 (19%) had 6 years of experience. 1 (4.8%) had 7 years of experience. 2 (9.5%) had 9 years of experience. 2 (9.5%) had 10 years of experience.

#### 3.1.1 Statistical analysis

**FIGURE 1:**
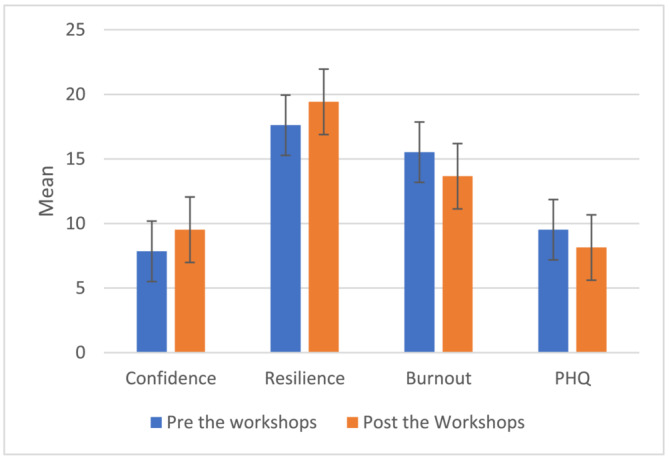
Mean levels of confidence in handling adverse events before and after attending the workshops.

Participants' confidence scores were higher after Reboot‐C (*M =* 9.52, *SD =* 0.42) than before (*M =* 7.85, *SD =* 0.40). This difference (−1.66) was significant, *t* (20) *= −*3.58, *p =* 0.002 *BCa*95*%* Cl [*−*2.47 *to −*0.90] (Figure 1). Participants' resilience levels were also higher on average after Reboot‐C (*M =* 19.42, *SD =* 0.72) than before (*M =* 17.61, *SD =* 0.76) (see Figure 1), and this difference (*−*1.80) was, again, statistically significant, *t* (20) *= −* 3.14, *p =* 0.005, *BCa*95*%* Cl [*−*2.91 to *−*0.85].

On average, participants' burnout levels were lower after Reboot‐C (*M* = 13.66, *SD* = 1.66) than before (*M* = 15.52, *SD* = 1.66). This difference (1.85) was statistically significant, (*t* (20) = 5.24, *p* = 0.000), *BCa*95% Cl [128, *−*2.42] (Figure 1).

The PHQ scores were lower in participants after Reboot‐C (*M* = 8.14, *SD* = 1.08) compared to before (*M* = 9.52, *SD* = 1.57) but this difference (1.38) was not statistically significant (*t* (20) =1.719, *p* = 0.101), *BCa*95% *Cl* [*−*0.14–3.00] (Figure 1).

#### 3.1.2 Feedback questionnaires

Most urology trainees said they strongly agreed or agreed that the workshops were relevant to their professional group (Table 2). Ninety percent of participants mentioned that they agreed or strongly agreed that the skills they learnt in the workshops would be useful in the future. Also, 90.4% of participants said that there was adequate time to cover the material in the workshops.

**TABLE 2:** Feedback questionnaire data.

	Strongly disagree	Disagree	Neither disagree or agree	Agree	Strongly agree
Q1. The workshops were relevant to my professional group	0	14.3% (3)	14.3% (3)	33.3% (7)	38% (8)
Q2. I learned skills in the workshops which will be useful in future	0	0	9.5% (2)	52.4% (11)	38.1% (8)
Q3. There was adequate time to cover the material	0	0	9.5% (2)	57.1% (12)	33.3% (7)
Q4. I found the workshops engaging	0	0	4.8% (1)	38.1% (8)	57.1% (12)
	**Yes**		**No**		
Q5. Were there any aspects of the workshops you did not find useful?	9.5% (2)		90.5% (19)		
Q6. Is there anything else you would have liked to see in the workshops which was not included?	19% (4)		81% (17)		
Q7. If you were involved in stressful workplace event, would you do anything differently as a result of attending this workshop?	71% (15)		19% (4)		
Q8. Would you recommend the training to other healthcare professionals?	90.5% (19)		9.5% (2)		

Regarding the question as to whether there were any aspects of Reboot‐C they did not find helpful, 19% said yes. When asked to specify, they mentioned, ‘I think it is tricky when being asked to give an example to not have enough time to dwell on it’. Regarding the question, is there anything missing from the workshops? 19% said yes; two participants mentioned they would like more breakout sessions to work through the scenarios with more breakout rooms, and two mentioned more information and practical tips.

When asked, ‘If you were involved in a stressful workplace event, would you do anything differently as a result of attending this workshop?’, 15 (71%) urology trainees said yes and mentioned the techniques they learned in the workshops, such as *‘*I would do the pie chart self‐care and postpone my worry*’* (supporting information Appendix S3).

Ninety percent of participants mentioned that they would recommend the training to other HCPs (Table 2). When asked to specify why they would recommend Reboot‐C, eight of them said they felt there was a need for workshops like this. For example, *‘*I think this is useful both for trainers and trainees*’*. Three participants said they recommended it because it is useful, for example, *‘*excellent material … applicable to all*’*.

The last part of the feedback questionnaire, ‘additional comments related to this training’ had seven positive comments, such as, *‘*For me, I will say that I'm grateful for the session. It was interactive*’*.

#### 3.1.3 Interview results

Thirteen urology trainees attended the interview, which took around 30 min (ranging from 20 to 42 min). Four themes emerged from the interviews: (1) deeper understanding, (2) providing a toolkit, (3) peer‐to‐peer interaction and engagement and (4) leaving them wanting more (Table 3).

**TABLE 3:** Example of themes and subthemes to evaluate the Reboot workshops on urology trainees.

Themes	Subthemes	Example
**Deeper understanding**	Raises awareness and personal understanding	I feel like I'm more able to recognize the negative thoughts and patterns and to try and overcome them by being aware of them and to have certain strategies. (Interview 3)
	Better understanding of other people	I think it helps my resilience and also, I think it helps me understand that of my team as well and how to support my team better. (Interview 4)
	Increased self‐esteem/more positive self‐view	I've been using the tools that we learned in the workshop about how to value the self in different ways (Interview 2)
**Reboot workshops as providing a toolkit**	Build a coping strategy toolkit/	I think with the help of resilience you'll be able to correct your past mistake and also, those things that you feel it was difficult to do in your life, you'll be able to do it like you will be able to make it easy. (Interview 8) I think it's more than skills, it's just helped make a path and steps to take. If you know when I do make a mistake that I can follow. (Interview 9)
	Gratitude diaries as a tool	There was a lot of helpful tools or strategies to manage negative habits. So like, practicing gratitude (Interview 1)
	Practicing postponing worry as a tool	If I'm operating, there is a mistake in one case, it will help me at least clear my mind by delaying the worry and focus on the next case. (Interview 2)
	Helps prepare surgeons for errors and AEs	We have either made errors or you know, difficult situations and I think this will definitely help me with previous events and future events. (Interview 6)
	Benefit of ‘homework’	Whole different coping mechanisms and how we would implement those and try to actually use it and see if that makes any difference. (Interview 6)
	Benefit of practical exercises	And those basic exercises that were done when just reinforced my understanding. (Interview 10)
**Peer to peer interaction and engagement**	Value of peer engagement and support	I like the fact that we worked through the examples together as a group. We had different people putting in different ideas and inputs about how they manage these situations. And also I liked how, as a group, we work through the cases to try and point out where the mistakes were, and where they can be rectified and how so. (Interview 9)
	Small group, more accessible interactions	I thought it was quite good that it was fairly small groups. I think we basically just had like eight or nine people, so I think that was quite good. (Interview 3)
	Benefits of online accessibility	I do like the Online. I think it is valuable. And it makes it easier to take up such an opportunity for example, particularly as something that's done on a regional scale, and maybe even had people from much wider than just the region of Yorkshire. But if people were traveling to such a workshop in person, you would spend, you could spend the same amount of time traveling as you do in the workshop, one hour there and one hour back, and it would reduce one's interest in going in person. (Interview 7)
	Disadvantages of doing it online/in person would encourage personal disclosures and peer‐to‐peer sharing	The only problem I have about online, I think is better, but the problem I had was during the focus group, you'd notice that some people were not responding. That will show that they weren't in the call. They were doing something else. They were listening and might just be there just for another reason. So that's, that's how I feel about the online session. So if it was more of in person than you'd see, you'd notice that people will be focus, they will listen, because then they're looking at the person but it's more online. So that's the only problem I have with online, but then when it comes to being comfortable when it comes to being accommodating, I'd say it's, it's really great. (Interview 12).
**Left wanting more**	Having one‐to‐one session with therapy/coaching.	‘I felt if I'm talking to one trainer or one lecturer talking to me will make me open’ (Interview 1).
	Extend the workshops with more details.	I feel there is still more to learn. If they could put in more hours, maybe make it up to three hours. So one could learn more, because from what I saw, the host was really time conscious. Most times It looked as if she was a rushing, um, so she could meet up on the two hours. So what I would say is they should put in more time to this sort of workshop because it is really helpful. (Interview 11)
	More breakout session	I think having more of a breakout session because I noticed on the second workshop, we did a breakout session. Where we disconnected from the right person, we went into the small subgroups, and suddenly the conversation flows a bit better. I don't know if people were just intimidated by having a larger group or having someone who's an expert in the area and they're worried. But I felt like the conversation was a bit better in that sense. I don't know if integrating that a bit earlier on might help people relax and engage a bit more. (Interview 5)

##### Deeper understanding

Urology residents who participated in Reboot‐C reported that they started to pay more attention to their thoughts, how they think and how their thinking affects their lives. Some stated that they had started to develop a greater personal understanding of their negative thoughts: *‘*I feel like I'm more able to recognize the negative thoughts and patterns and to try and overcome them by being aware of them and to have certain strategies*’* (Interview 3).

The urology residents felt Reboot‐C boosted their social support and increased their capacity for empathy: *‘*I think it helps my resilience, and also, I think it helps me understand that of my team and how to support my team better*’* (Interview 4).

Participants finished Reboot‐C with a more positive outlook on themselves and experienced a boost to their sense of self‐worth: *‘*I've been using the tools that we learned in the workshop about how to value the self in different ways*’* (Interview 2).

##### Providing a toolkit

This theme describes how Reboot‐C helped urology trainees build a coping strategy toolkit. Many urology trainees mentioned that Reboot‐C made them more comfortable with the methods they employ to deal with everyday challenges. For example, one trainee mentioned that *‘*I think it's more than skills, it's just helped make a path and steps to take. If you know—when I do make a mistake—that I can follow’ (Interview 9).

One of the techniques urology trainees learned in Reboot‐C was the practice of regularly writing down and thinking about the things they were grateful for: ‘There were a lot of helpful tools or strategies to manage negative habits. So, like, practising gratitude’ (Interview 1). In addition, one of the strategies trainees learned was practising postponing worry. This is a technique for managing emotion by deciding a specific time to worry and ‘postponing’ worries up to that point by instead writing them down and mentally moving on. As one trainee said, ‘If I'm operating, there is a mistake in one case, it will help me at least clear my mind by delaying the worry and focus on the next case’ (Interview 2).

Urology trainees said they benefited from ‘homework’; Reboot‐C suggested surgeons plan to do the technique they had selected in between workshops as homework. One urology trainee said they felt that they had learned *‘*whole different coping mechanisms and how we would implement those and try to actually use it and see if that makes any difference*’* (Interview 6). Urology trainees also benefitted from the practical nature of the exercises, *‘*And those basic exercises that were done… just reinforced my understanding*’* (Interview 10).

##### Peer‐to‐peer interaction and engagement

One of the perceived strengths of Reboot‐C was peer‐to‐peer interaction and engagement, which made the urology trainees more open to sharing their ideas and the difficulties they were facing.

Working with a small group made participation easier; many urology trainees noted that this made workshops more approachable for interacting with colleagues and sharing information in a more casual and relaxed setting, *‘*I thought it was quite good that it was fairly small groups. I think we basically just had like eight or nine people, so I think that was quite good*’* (Interview 3).

Some urology trainees mentioned the benefit of the online format, which made participating in Reboot‐C more accessible and allowed them to meet a variety of people: *‘*I do like the online. I think it is valuable. And it makes it easier to take up such an opportunity, for example, particularly as something done on a regional scale and maybe even had people from much wider than just the region of Yorkshire. But if people were travelling to such a workshop in person, you would spend, you could spend the same amount of time travelling as you do in the workshop, one hour there and one hour back, and it would reduce one's interest in going in person*’* (Interview 7).

However, some others saw disadvantages of doing it online as they thought in‐person workshops would encourage personal disclosures and peer‐to‐peer sharing: ‘The only problem I have about online, I think is better, but the problem I had was during the focus group, you'd notice that some people were not responding. That will show that they weren't on the call’ (Interview 12).

##### Left wanting more

Some participants believed that the intervention should be longer, for example, by including one‐to‐one sessions with the therapist delivering the workshops. For example, one urology trainee mentioned, *‘*I felt if I'm talking to one trainer or one lecturer talking to me will make me open*’* (Interview 1).

One suggestion was to extend the workshops by an hour or more to provide more information. For example, ‘there is still more to learn. If they could put in more hours, maybe make it up to three hours. So, one could learn more because, from what I saw, the host was really time conscious. Most times, it looked as if she was rushing, um, so she could meet up on the two hours. So, I would say they should put more time into this sort of workshop because it is really helpful*’* (Interview 11). Others mentioned that they wanted more breakout sessions, to make the conversation flow a bit better. For example, *‘*I think having more breakout sessions*’* (Interview 5).

In summary, it can be clearly seen that, when evaluating Reboot‐C by interviewing urology trainees, most of the trainees described experiencing a positive outcome of their participation, such as a deeper understanding of their personal mental health. Providing a toolkit, peer‐to‐peer interaction and engagement made the urology trainees more open to sharing their ideas and the difficulties they were facing, and they also left wanting to know more about the Reboot‐C techniques (see the supporting information Appendix S4 for the whole representative quotes from the Interviews).

## 4 DISCUSSION

Trainees who participated in Reboot‐C saw significant improvements in their resilience and confidence levels and a significant reduction in burnout. However, there was no substantial reduction in depressive symptoms. The intervention components had high retention, with the majority of participants completing the two workshops and the questionnaires that were collected immediately after these. Regarding written feedback, most trainees found Reboot‐C engaging, useful and relevant to their profession. Some trainees reported that they wanted more breakout sessions to work through the scenarios, with more breakout rooms and more information regarding practical tips. Analysis of interview data indicated that Reboot‐C helped urology trainees develop a deeper understanding of their mental health and provided a toolkit of skills for enhancing resilience. They found the workshops also useful for peer‐to‐peer interaction and engagement, and the workshops left them wanting more. Overall, the evaluation indicated that Reboot‐C was acceptable to urology trainees.

Previous Reboot studies have focused on nurses^23^ and multidisciplinary HCPs and students.^19^ This study is the first to investigate Reboot in urology trainees. Furthermore, this is the first study to investigate Reboot‐C, the version of Reboot that does not include a one‐to‐one coaching session with the facilitator. Our findings demonstrate that Reboot‐C, a lower‐resource intervention, can provide benefits to participants. As the study investigates a potential burnout reduction intervention among urology trainees, this work is timely. Burnout is particularly high in surgical trainees, but only a limited number of original interventional studies have been published in this group.^19^

Reboot‐C could play an essential role in improving urology trainees' ability to prepare for work‐related difficulties, such as the post‐operative complications of patients and adverse events. Reboot‐C might also help raise urology trainees' awareness of burnout, thus improving their mental health, specifically to deal with complications. Turner et al. found that surgeons' emotional reactions to unforeseen complications typically surprised them. Half of the 445 surgeons polled had moderate to severe anxiety, 30% had difficulties sleeping, 33% had anger or irritation issues and 10% were depressed.^33^ Yet, Reboot‐C is the first prophylactic intervention to be tested in urology trainees. Raising awareness of burnout can be essential for reducing the urge to suffer in silence, as it is typical for healthcare workers to remain silent regarding medical‐related errors.^34^

However, the present study had several limitations. First, it only measured the short‐term effects of Reboot‐C, and there is a need for more research that measures the long‐term effects. Second, this study had a small sample size. While this is less of a limitation regarding the interviews, which included 13 participants providing rich data, caution should be taken when interpreting the results of the quantitative results. Third, the study did not include a control group. According to Wiederhold et al (2018), who conducted a systematic evaluation of interventions for physician burnout, many interventional trials have methodological flaws. In particular, most studies lack randomised control groups.^35^

There is a need to evaluate interventions with the highest probability of being effective,^31^ and the current findings indicate that Reboot‐C could be a useful candidate intervention for more extensive controlled trials in the future. Also, the current research focuses only on urology trainees' resilience, not the work environment to reduce burnout. There is a need for more research focusing on a strategy based on determining the best ‘fit’ between surgeons and their work environments.

### 4.1 Implications

Many interventions are one‐on‐one, and as a result, they require a significant investment of both time and resources. Despite this, there is an urgent need for more effective and acceptable interventions to reduce burnout. This intervention only requires 4 h of time from trainees, and it may be provided remotely or in groups, making it easier to access and less resource demanding. It is possible for training programmes to make Reboot available to their participants. However, the results of this study do not provide definitive conclusions regarding intervention effectiveness. Instead, the results should be regarded with caution. This is only a preliminary study, and a larger, controlled study is needed.

## 5 CONCLUSION

This study evaluated Reboot‐C for urology trainees and found that it was acceptable and associated with improved resilience, confidence and burnout levels. This study's findings pave the way for more extensive studies on the efficacy of Reboot‐C for surgeons.

## AUTHOR CONTRIBUTIONS

T. G., J. J., C. S. B. and D. O. C. were responsible for designing and implementing the research, analysing the results and writing the manuscript. R. C. and J. J. delivered the workshops, and all authors provided feedback on the manuscript draft.

## CONFLICT OF INTEREST STATEMENT

None of the authors has a conflict of interest to declare.

## Supporting information


**Appendix S1.** Supporting Information
**Appendix S2.** Supporting Information
**Appendix S3.** Supporting Information
**Appendix S4.** Supporting Information
