# Supplementary material for: Evaluation of the reboot coaching workshops among urology trainees: A mixed method approach
Source: BJUI Compass. 2023 May 2;4(5):533–42. doi: 10.1002/bco2.249 (PMC10447217; doi:10.1002/bco2.249)
Supplement: Supplementary file 1 — Appendix S1. Supporting Information Appendix S2. Supporting Information Appendix S3. Supporting Information Appendix S4. Supporting Information [file BCO2-4-533-s001.docx]

**Supplementary file**

**Appendix A**

**Baseline questionnaire and follow-up (for intervention group)**

**Unique identifier creation:**

| Last 2 digits of your mobile phone number (e.g., ‘95’) |  |
| --- | --- |
| Day of the month on which you were born (e.g., ‘14’) |  |
| Third letter of your mother’s surname (e.g., ‘a’) |  |

**Questionnaire proforma**

1. What is your age?

21-30 □

31-40 □

41-50 □

51-60 □

61-70 □

Prefer not to say □

1. What is your gender? Male □ Female □ Other □ Prefer not to say □
2. What is your ethnic group?

| **White** | | **Black or Black British** | | | | |  |
| --- | --- | --- | --- | --- | --- | --- | --- |
| British |  | African | | | |  |  |
| Irish |  | Caribbean | | | |  |  |
| Any other White background |  | Any other Black background | | | |  |  |
| **Asian or Asian British** | | **Mixed** | | | | |  |
| Indian |  | White and Black Caribbean | | | |  |  |
| Pakistani |  | White and Black African | | | |  |  |
| Bangladeshi |  | White and Asian | | | |  |  |
| Any other |  | Any other mixed background | | | |  |  |
| **Other Ethnic Group** | |  | | | | |  |
| Prefer not to say |  |  |  | | | |  |
| Any other (please specify) |  | | |  |  | | |

1. Please indicate which stage of training you are at:
   1. ST1
   2. ST2
   3. ST3
   4. ST4
   5. ST5
   6. ST6
   7. ST7
   8. Other………….
2. How many years have you been qualified? [open response]___________________________

|  | No – not at all | Unsure | Yes – to some extent | Yes - definitely |
| --- | --- | --- | --- | --- |
| If I was involved in a serious adverse event for which I thought I held some responsibility, I am confident I’d know where to look for practical help/tips | 1 | 2 | 3 | 4 |
| If I was involved in a serious adverse event for which I thought I held some responsibility, I am confident I’d know where to go for emotional support | 1 | 2 | 3 | 4 |
| If I was involved in an adverse event for which I thought I held some responsibility I know the things I would do to help manage my stress levels | 1 | 2 | 3 | 4 |

|  | Strongly Disagree | Disagree | Neutral | Agree | Strongly Agree |
| --- | --- | --- | --- | --- | --- |
| I tend to bounce back quickly after hard times | 1 | 2 | 3 | 4 | 5 |
| I have a hard time making it through stressful events | 1 | 2 | 3 | 4 | 5 |
| It does not take me long to recover from a stressful event | 1 | 2 | 3 | 4 | 5 |
| It is hard for me to snap back when something bad happens | 1 | 2 | 3 | 4 | 5 |
| I usually come through difficult times with little trouble | 1 | 2 | 3 | 4 | 5 |
| I tend to take a long time to get over set-backs in my life | 1 | 2 | 3 | 4 | 5 |
| On the whole, I am satisfied with myself | 1 | 2 | 3 | 4 | 5 |
| At times I think I am no good at all | 1 | 2 | 3 | 4 | 5 |

**Burnout items (items with the highest factor loadings on each subscale of the Oldenburg Burnout Inventory; OLBI; Demerouti et al., 2007/8).**

The response scale is from ‘1’ (strongly agree) to ‘4’ (strongly disagree).

D4: Challenging: ‘I find my work to be a positive challenge’

D1: Interesting aspects: ‘I always find new and interesting aspects in my work’

D8: More engaged: ‘I feel more and more engaged in my work’

E6: Worn out: ‘After my work, I usually feel worn out and weary’

E2: Longer times for rest: ‘After work, I tend to need more time than in the past in order to relax and feel better’

E5: Fit for leisure activities: ‘After working, I have enough energy for my leisure activities’


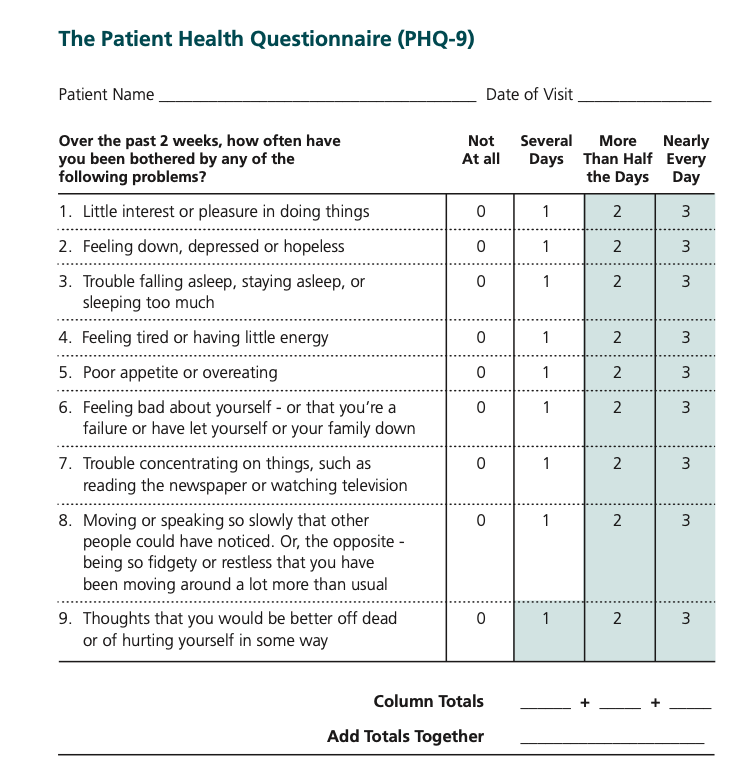


**Feedback evaluation form (for follow up)**

Questions 1-4: please circle your response to indicate the extent to which you agree with the statements

Q1.The workshops were relevant to my professional group

| Strongly disagree | Disagree | Neither disagree or agree | Agree | Strongly agree |
| --- | --- | --- | --- | --- |

| Strongly disagree | Disagree | Neither disagree or agree | Agree | Strongly agree |
| --- | --- | --- | --- | --- |

Q2 I learned skills in the workshops which will be useful in future

Q3 There was adequate time to cover the material

| Strongly disagree | Disagree | Neither disagree or agree | Agree | Strongly agree |
| --- | --- | --- | --- | --- |

Q4 I found the workshops engaging

| Strongly disagree | Disagree | Neither disagree or agree | Agree | Strongly agree |
| --- | --- | --- | --- | --- |

Q5 Were there any aspects of the workshops you did **not** find useful? Yes/No

If YES please describe these here:


Q6 Is there anything else you would have liked to see in the workshops which was not included?
Yes/No
Please describe:

Q7 If you were involved in stressful workplace event, would you do anything differently as a result of attending this workshop?
Yes/No
Please describe:

Q8 Would you recommend the training to other healthcare professionals?

Yes/No

Please describe:


Please add any additional comments related to this training here:

**Appendix B**

**Interview schedule (as per Johnson et al, 2020)**

**Promoting psychological resilience in urology trainees**

**Topic Guide - Follow-up Interviews with Participants**

**RESILIENCE and ERROR MANAGEMENT**

1. Can you describe to me your understanding of the concept of resilience?
   1. Had you come across this concept prior to the online workshops?
   2. Do you feel that you have a good understanding of the concept after taking part in the workshops?
2. To what extent do you feel that higher levels of resilience may help you in your professional practice?
3. Since engaging in the online workshop, how do you feel about your ability to cope with instances of error arising during your work?
4. To what extent do you think that being more resilient may help you to manage instances in which you have made a mistake in the course of your work?
   1. In what ways, if any, do you think that being more resilient may help you?
   2. Do you feel that the workshop has provided you with any useful skills for such instances?
5. To what extent do you feel that the skills developed are relevant and useful to your future career as a health professional?

**WORKSHOP and follow up phone-call/tutorial**

I’d now like to ask you a bit about your experience of the workshop.

1. What was your overall perception of the workshop?
   1. Probe particular issues arising here
2. What did you think worked well?
   1. Probe particular issues arising here
3. What could be improved?
   1. Probe as to how improvements might be made
4. How did you feel about the online format?
5. How did you find discussing these issues in a group?
6. How did the workshops contribute to your learning and overall professional development in relation to resilience?
7. Overall, would you recommend this session to others undertaking your professional training?

*That brings us to the end of the question I have for you but do you have anything you would like to add or any questions for us? Thank the participant.*

**Appendix C**

***If you were involved in a stressful workplace event, would you do anything differently as a result of attending this workshop?***

| If you were involved in a stressful workplace event, would you do anything differently as a result of attending this workshop?   1. Use the techniques learnt to not beat myself up after stressful events. 2. Useful to consider shared responsibility, healthy coping behaviours. 3. Assertive communication. Not dealing with it. 4. Take time out to understand the situation better in order to give an appropriate response. 5. I have some strategies to help manage the stress of that situation 6. Yes, I would handle it myself. 7. Paying more attention to how I plan the rest of the day in order to allow myself to "de-stress". 8. I would do the pie chart, Self-care and postpone my worry. 9. Yes, inquiry and doing something I have learnt from the workshop. 10. Use short-term and long-term strategies. Make a pie chart on blame assertion. 11. Breathing exercises and trying to stop the negative thoughts by counteracting them with more positive interpretation. 12. Apply more of the postponing worry. 13. Avoid discounting the positive. 14. Take more time to focus on some of the contributing factors. Reflect on positive events to boost self-confidence. 15. Be better equipped to recognise your own behaviours and tendencies and hence be able to break the cycle. |
| --- |

**Appendix D**

**Themes, subthemes, and representative quotes from the Interviews:**

1. **A Deeper Understanding**

| **Subtheme** | **Quotes** |
| --- | --- |
| **Raises awareness and personal understanding** | “What I understand from the workshop is that it's like what we call our habit and method of thinking that we could implement in order to break the cycle of negative thinking and minimise the cycle of cognitive behaviour when triggers happen in our emotions. Turn to your thoughts” (Interview 1).  “I feel like I'm more able to recognise the negative thoughts and patterns and to try and overcome them by being aware of them and having certain strategies” (Interview 3).  “And I think I understand your way of thinking, so I think I ruminate quite a lot” (Interview 6).  “I think putting that into a sort of category of what that activity is part of a negative thought pattern. So, then, you can cope with it better by recognising what you're doing. I don't know if that makes sense” (Interview 6).  “I feel like I'm more able to recognise the negative thoughts and patterns and try and overcome them by being aware of them and having certain strategies.” (Interview 3).  “The most important thing is to be able to recognise the situation and process it properly. So, recognising women and negative events happening, and then trying to sort of adapt their thought processes and to try and break that cycle of negative emotions and behaviours” (Interview 3).  “I believe I don't have a good understanding of it, and the workshop has helped as well” (Interview 7).  “Yeah, with the help of the workshop. I learned so many things, like mental health, this and cognitive behaviour” (Interview 8).  “It has been easy, like the workshop also gave me a clearer  understanding, and it makes it more easy for me” (Interview 8).  “I think it's more than skills; it's just helped make a path and steps to take. If you know when I do, make a mistake that I can follow " (Interview 9).  “I've come across them, but the workshop brought more light and enlightenment to the term describing what it really is.” (Interview 11).  “I have a proper understanding after taking part in the workshop, because it was really elaborate on that.” (Interview 12).  “So, that was my perception about the workshop that was kind of realized, and I've really been able to acquire more knowledge. But many things I didn't really know you've got to learn. (Interview 13). |
| **Better understanding of other people** | “I think it helps my resilience, and I also think it helps me understand that of my team and how to support them better.” (Interview 4).  “I think it will help in dealing with a difficult interaction, either with patients or colleagues.” (Interview7).  “Also, how to relate to people, how to communicate with people to help you. Really helpful” (Interview 8).  “Especially when it comes to care of social behavioural” (Interview10).  “I've learned from psychological communication and physical and emotional well-being, and you know, I can have emotions from different people and learn how to resist and communicate with them.” (Interview 13). |
| **Increased self-esteem/more positive self-view** | “I've been using the tools that we learned in the workshop about how to value the self in different ways” (Interview 2).  “So, I've started now using the fact that when I get positive feedback, I don't usually discount the positive, which I used to do frequently” (Interview 2). |

1. **Reboot Workshops as Providing A Toolkit**

| **Codes** | **Example** |
| --- | --- |
| **Build a coping strategy toolkit/** | “I have more resilience. I would be facing issues easier than before.” (Interview 1).  “Of course, when something happens, we all have emotions, but I just thought I was talking about the thoughts I keep. I keep thinking about the same thing. I keep thinking about the negative thought, and this actually reflects on my behaviour at work. For example, I was involved in a complaint for a patient, and since I received the email to give my statement regarding this complaint, I was worried about missing something documented in the notes. So since then, I've been documenting anything in the notes, even unnecessary things. So, this is bad behaviour, and it was obvious to one of my colleagues that I changed my writing after the complaint. So, if I applied this to that example, I should stand here if I'm more resilient; it will not affect the behaviour more. I should stop thinking about myself as if I'm missing things. So, I am going to the other extreme and writing any unnecessary things, you know, what I'm talking about, to stop the cycle. So, this is how I understand it” (Interview 1).  “Better understanding and more tools to establish resilience” (Interview 2).  “How I've been using them, the way that they were phrased in three different categories, is quite useful, and I'm trying now to integrate them into one category, like an activity from each category on its own, in my every day to boost my mood, with some success and some failure. So, it's a bit of a trial-and-error kind of thing. So, getting through that” (Interview 2). “I'm trying to get my mood better by using the skills that I've learned” (Interview 2).  “I'd be more in control of the situation in terms of if something were to happen rather than sort of having the negative thoughts, it's more, I feel like I'm more able to recognise the negative thoughts and patterns and try and overcome them by being aware of them and having certain strategies” (Interview 3).  “These workshops have helped me to have a bit more of an understanding of what sort of factors can cause mistakes and have better coping strategies” (Interview 3).  “As far as these workshops go, these workshops have helped me to have a bit more of an understanding of what sort of factors can cause mistakes and have better coping strategies." (Interview3).  “Understanding of resilience. I think initially, we always think resilience is about how to perform better, but this is obviously not the truth. It's more about how you cope better. (Interview 6).  “In reframing one's thought process and not dwelling on only the negative aspects of a scenario” (Interview 7).  “I think with the help of resilience, you'll be able to correct your past mistake and also, those things that you feel it was difficult to do in your life, you'll be able to do it like you will be able to make it easy” (Interview 8).  “It has been easy, like the workshop also gave me a clearer understanding, and it makes it more easy for me” (Interview 8).  “I think with the help of resilience, you'll be able to correct your past mistake and also, those things that you feel it was difficult to do in your life, you'll be able to do it like you will be able to make it easy.” (Interview 8).  “I think it is like the capacity to recover something quickly” (Interview 8). “I think it's more than skills; it's just helped make a path and steps to take. If you know when I do make a mistake, I can follow your lead” (Interview 9).  “I think it's more than skills; it's just helped make a path and steps to take. If you know when I do make a mistake, I can follow your lead” (Interview 9).  “It would just help me give almost a path or guide on what to use when I make mistakes or when, say, my trainees or my colleagues make mistakes as to how to guide them through the time where it's quite difficult.” (Interview 9). |
| **Reboot as improve – gratitude diaries as tool of the reboot workshop** | “There were a lot of helpful tools or strategies to manage negative habits. So, like, practising gratitude (Interview 1)  “Now I try to keep a diary of things that I'm kind of thankful for, and when I was thinking about it, this is basically what I found to be an Islamic concept. So, in a way, it's like, "Just count your blessings, and they'll become more plentiful," so I've been trying to do more of that. (Interview 2).  “I think the workshop helped with how I applied some of the exercises that were recommended, like reframing one's negative thoughts and also trying to use a gratitude exercise to think more about the positive” (Interview 7).  “In practising gratitude on a daily basis” (Interview 7). “One of them is practising being thankful. Putting up a positive mindset and finding a distraction” (Interview 11). |
| **Practicing postpone worry as Reboot tool** | “There were a lot of helpful tools or strategies to manage negative habits. So, like, practising gratitude or losing worry (Interview 1).  "If I'm operating and there is a mistake in one case, it will help me at least clear my mind by delaying the worry and focusing on the next case” (Interview 2).  “I could adapt; even when there's a catastrophe, it will really help me because I don't have to look at the mistakes for so long. I don't have to take it to heart. I don't have to be bothered by it all the time” (Interview 12).  “First of all, keep the mindset. I'm straight. Making sure you don't let yourself being bothered by mistakes because they often do happen. But then you need to stay focused. I think being resilient could really help me in that way. Because I have a profession that I need to carry on. So I don't need to look back. I don't need to be distracted; I just need to stay focused and know how to go about whatsoever may be a boredom or border” (Interview 12). |
| **Helps prepare surgeons for errors and AEs.** | “Of course, when something happens, we all have emotions, but I just thought I was talking about the thoughts I keep. I keep thinking about the same thing. I keep thinking about the negative thought, and this actually reflects on my behaviour at work. For example, I was involved in a complaint for a patient, and since I received the email to give my statement regarding this complaint, I was worried about missing something documented in the notes. So since then, I've been documenting anything in the notes, even unnecessary things. So, this is bad behaviour, and it was obvious to one of my colleagues that I changed my writing after the complaint. So, if I applied this to that example, I should stand here if I'm more resilient; it will not affect the behaviour more. I should stop thinking about myself as if I'm missing things. So, I am going to the other extreme and writing any unnecessary things, you know, what I'm talking about, to stop the cycle. So, this is how I understand it. (Interview 1).  “Even if the mistake may have been mine, there's a lot of other factors—reload stress, etc.—and communication between members of the team. And then, if it's obviously a mistake, it's a case of reflecting on it and trying to learn from it, and trying to avoid the negative habits like stressing and ruminating and personalising it, and sort of having repetitive actions, and sort of being more aware of the whole process of making mistakes and what things to watch out for, and the aftermath of making a mistake.” (Interview 3).  “It's more about, you know, having the necessary capacity to try and adapt to and having a structured framework for managing adversity” (Interview 3).  “When someone's had something go wrong, and then trying to identify what sort of processes might occur in terms of how they might deal with it and how to actually deal with it and how to structure based on actual case scenarios is quite useful” (Interview 3).  “I suppose it'd be being able to cope and continue to perform in, you know, an adverse environment or following an adverse event” (Interview 4).  “It might help you cope better with acknowledging when a mistake has happened and being able to move forward past that mistake” (Interview 5).  “It's been more resilient to try and avoid making mistakes because of adverse events" (Interview 4).  “I do appreciate having them tailored to a certain specialty, particularly when you're discussing adverse events" (Interview5).  “ I think it does help in the sense that if you've made a mistake” (Interview 6).  “We have either made errors or, you know, been in difficult situations, and I think this will definitely help me with previous events and future events” (Interview 6).  “I have personally experienced stressful situations, and I think that helped me put things into perspective, especially when you watch that anaesthetic video about that. That guy made a mistake, but it wasn't really him that made a mistake, but I think everyone always feels like the onus is on that. I think that was really useful” (Interview 6).  “I think it will help in dealing with difficult interactions, either with patients or colleagues, and with difficult or negative experiences in the workplace and in one's professional career.” (Interview7). “I think I'd better be able to cope with errors arising” (Interview 9).  “I think it is very important to be able to manage and move on from mistakes” (Interview 9).  “What I learned from the workshops is that errors are something that occurs, are not something that is prepared for, are something that most times you may not know, you did not know it just happens, surprisingly, without your knowledge, so that is what I understand about errors. But what I learned is how to get out of the mindset and the stigma that comes with correcting errors” (Interview 11).  “Being able to manage failures and all of that, so that was one way that I'm able to think about being resilient” (Interview 11).  “Make sure you don't let yourself be bothered by mistakes because they often do happen. But then you need to stay focused. I think being resilient could really help me in that way. Because I have a profession that I need to carry on. So I don't need to look back. I don't need to be distracted; I just need to stay focused and know how to go about whatsoever may be a boredom or border” (Interview 12). |
| **Benefit of ‘homework’** | “Whole different coping mechanisms and how we would implement those and try to actually use them and see if that makes any difference” (Interview 6).  "Also, the various sections where we could impute some words into our book, that was great. So if I feel I'm not getting something right, I could make reference to the workbook. I could look out. Oh, this is what I wrote. And this is what I was doing. So I won't say to that person, "It'll really help” (Interview 12).  “I'll always make reference to the workbook because that's what I really liked. You know, having to put our ideas down. So, like I said, for reference purposes, what you practise before the next workshop—that was one of the texts that was really great. For me, I practised that practise of distraction by trying to distract myself when I tried to get bothered” (Interview 12). |
| **Exercise to applying resilience / Benefit of practical exercises** | “Practicing the exercises was necessary because if you talk about theories, I feel like I know them already, but my knowledge was not reflected in my actions, so it took to apply the theory in practise and give you a week to then rediscuss it again. Yeah. Practicing the exercises was nice” (Interview1).  “I have used some of the skills that I've gained in the workshop throughout my day” (Interview 2).  “A good thing in the workshop is to try and find more tools on how to do that” (Interview 2).  “When someone's had something go wrong, and then trying to identify what sort of processes might occur in terms of how they might deal with it and how to actually deal with it and how to structure based on actual case scenarios is quite useful” (Interview 3).  “I think from that side of things, learning more about resilience has been very useful in my professional work in the future.” (Interview 3).  “I think it's one of the more interesting exercises for me; it was just to see how far you could share the blame. I suppose it's all about blame, but just how far can you sort of spread responsibility across many different people in many different positions for anything that may happen?” (Interview 4).  “And what's useful for some people might not be useful for a difference. So, I think it's very relevant to go through all of them. Because then each person can identify what's more useful for how they work” (Interview 5).  “I think it's good to go over techniques to fight the negative thinking or identify different types of negative thinking as well. I think going over all these things again was quite useful for me” (Interview 5).  “We did the exercises, which was very useful because it allowed you to use the ideas and concepts in sort of real life, and I liked the fact that we did the whole different coping mechanisms and how we would implement those and try to actually use them and see if that made any difference” (Interview 6).  “I think the exercises worked really well. And I think when we did” (Interview 6).  “I think the workshop helped with how I applied some of the exercises that were recommended, like reframing one's negative thoughts and also trying to use a gratitude exercise to think more about the positive” (Interview 7).  “And the search made the case scenarios relevant and realistic” (Interview 7).  “But throughout the week, I've been using the tools that we learned in the workshop about how to value the self in different ways rather than just focusing on one aspect, which is work or relationships or physical appearance or whatever” (Interview 2).  “And those basic exercises that were done just reinforced my understanding.” (Interview 10).  “And we were asked to put in some write-ups in our workbook about practising a positive mindset” (Interview 11).  “The speaker was also great in trying to find out what to do, giving us some activities and the course of the workshop. So we could gather more understanding" (Interview 12).  “There's a lot of information, a lot of knowledge to be learned, a lot of new words that you didn't know, and a lot of exercises, so I definitely recommend it” (Interview 12).  “There's a lot of information, a lot of knowledge to be learned, a lot of new words that you didn't know, and a lot of exercises, so I definitely recommend it” (Interview 13).  “I think the process of interaction and, you know, the process of doing a different exercise and communicating to each other works very well and better” (Interview 13). |

1. **Peer to Peer Interaction and Engagement**

| **Codes** | **Example** |
| --- | --- |
| **value of peer engagement and support** | “Being in a group is better; we have it because we have the same exercise, but each one of us talks about another perspective, a different perspective. So, we open ideas to each other. (Interview 1).  “It gives you an idea of how different people understand a certain situation, even though they're reading the same script. Each person views it from a different perspective. So, it's quite important to know that. (Interview 2).  “So that exchange of ideas with colleagues, I think, is also particularly helpful” (Interview 5).  “I found them useful, as I said, being able to exchange ideas with your colleagues and having people say, 'Actually, I have the exact same concerns, fears, or problems, and this is what's worked'” (Interview 5).  “In the workshop, people brought up mindfulness during mental health and said that it really helped by, for example, reading theory books, playing games, running, and jogging, and that it really helped reduce mental health stress and improve mental health.” (Interview 8).  “And also, I really like to relate to people. to "talk to people that also work well.” (Interview 8).  “I prefer group calls because you'll be able to learn from other people's ideas” (Interview 8).  “I like the fact that we worked through the examples together as a group. We had different people putting in different ideas and inputs about how they manage these situations. And also, I liked how, as a group, we work through the cases to try and point out where the mistakes were, where they can be rectified, and how so” (Interview 9).  “I think the process of interaction and, you know, the process of doing a different exercise and communicating to each other works very well and better” (Interview 13).  "Share your ideas and get some more to share” (Interview 13). |
| **Small group, more accessible interactions** | “I thought it was quite good that there were fairly small groups. I think we basically just had like eight or nine people, so I think that was quite good” (Interview 3).  “There was a little bit of teamwork." So, it was a nice mix of didactic teaching with a bit of group work as well to work, and so I was -- I think it's yeah, it was a nice balance” (Interview 4).  “I thought it was the most comfortable setting. It was kind of like everything was going through as confidential in the session. In a larger group, people would be even more unwilling to share their experiences and things. It was a good size” (Interview 3).  “Actually, the most useful part is that it's a small group of people and you are forced to interact. So that was actually very useful. I think that that sort of eliminated the need and made it a bit more intimate” (Interview 6).  “I prefer group calls because you'll be able to learn from other people's ideas” (Interview 8).  “We had different people putting in different ideas and inputs about how they manage these situations. And also, I liked how, as a group, we worked through the cases to try and point out where the mistakes were, where they could be rectified, and how so” (Interview 9).  “I liked the fact that we were in a group setting and other people could voice their ideas and experiences, so I thought that was good” (Interview 9).  “I'd say it was also marvellous, having people bring in different mindsets, different presidents perspectives on different understandings. It was really great because you could just learn from the host. People could also say something that can bring light, like, Oh, this is something that was great. I must give it to the group members” (Interview 11). |
| **Benefits of online - accessibility** | “Nowadays, even if face-to-face would be better, I would not be able to attend. So, to talk about real life online is like more resilient. I can attend from home. So, after the work, I think we started at six and finished at 5:30 almost, so yeah, you know if it's face-to-face, I would not be able to attend“ (Interview 1).  “I think, but more than being more virtual, we add people from all over the place and more different experiences." (Interview4)  “But accessibility-wise, I understand why online courses are useful, and I think it's still a useful course” (Interview 6).  “I do like the online. I think it is valuable. And it makes it easier to take up such an opportunity, for example, particularly as something that's done on a regional scale and maybe even has people from much wider than just the region of Yorkshire. But if people were travelling to such a workshop in person, they would spend, they could spend the same amount of time travelling as they do in the workshop, one hour there and one hour back, and it would reduce one's interest in going in person” (Interview 7).  “I like to do online workshop because it's not everybody that we'll have time to go in person that they only show you can sit down at your convenience and join it” (Interview 8).  “But I think you would struggle to actually get people enrolled in it as much as online.” (Interview 9).  “The online was really great. Because I think most folks get access to that” (Interview 10).  “For me, being online is good because, as you know, not everybody is available to come. So from online, you know, at your conference or in your home or office, you can attend a workshop” (Interview 10).  “I think the online is better because people can sit in from the comfort of their own homes and attend the meeting” (Interview 11).  “You can sit down and, you know, login to your mobile phone, the laptop, or a gadget to attend the workshop” (Interview 13). |
| **Disadvantages of doing it online/in-person would encourage personal disclosures and peer-to-peer sharing** | “I suppose you'd have more of a conversation if it were face-to-face, but that's less practical, you know, in terms of what you're doing, but I think it works very well" (Interview 4).  “I think if it were face-to-face, it would probably naturally make it easier. Because I think the online aspect of it does create a bit of distance, even though I think it's easier to talk about these things in a room with a closed door rather than online, and people feel more comfortable when you can see each other, I guess” (Interview 6).  “I think the fact that if it were face-to-face, a workshop would be much better because you would actually get to see the people that you're with and you'll be a lot more engaged than if it were online” (Interview 9).  “But in our group, it was very much, and no one really spoke; everyone just used the textbox to message each other, and no one had the cameras turned on” (Interview 9).  “The only problem I have with online is that I think it's better, but the problem I had was during the focus group, when you'd notice that some people were not responding. That will show that they weren't on the call. They were doing something else. They were listening and might just be there for another reason. So that's how I feel about the online session. So if it were more in person than you'd see, you'd notice that people will be focused, they will listen, because then they're looking at the person, but it's more online. So that's the only problem I have with online, but then when it comes to being comfortable and accommodating, I'd say it's really great” (Interview 12). |

1. **Left Wanting More**

| **Codes** | **Example** |
| --- | --- |
| **Having One to one session with therapy/coaching.** | “I'm not quite sure if this is applicable or not. But if something like this—I know it's not psychological therapy or something like this—when they asked us to think in groups of three and three, and we did the exercise like this I felt that if I'm talking to one trainer or one lecturer, talking to me only be more open” (Interview 1).  “If you know it's difficult, but if you have a one-on-one discussion, I think this will be a bit better. I know it's difficult, but this is my only feeling” (Interview 1). |
| **Extend the workshops with more details.** | “The fact there were only two hours per session” (Interview 9).  “I think it could be a bit longer and a bit more detailed” (Interview 9).  “I feel there is still more to learn. If they could put in more hours, maybe they could make it up to three hours. So one could learn more because, from what I saw, the host was really time conscious. Most times, it looked as if she was rushing, um, so she could meet up within the two hours. So what I would say is that they should put in more time for this sort of workshop because it is really helpful” (Interview 11).  “I think the information in the workshop should be more practical” (Interview 13). |
| **More breakout session** | “I think we should have had more of a breakout session because, as I noticed on the second workshop, we did a breakout session. Where we were disconnected from the right person, we went into the small subgroups, and suddenly the conversation flowed a bit better. I don't know if people were just intimidated by having a larger group or by having someone who's an expert in the area and they're worried. But I felt like the conversation was a bit better in that sense. I don't know if integrating that a bit earlier on might help people relax and engage a bit more” (Interview 5).  “I think having more of a breakout session” (Interview 5). |
